# Towards standardizing basophil identification by flow cytometry

**DOI:** 10.3389/falgy.2023.1133378

**Published:** 2023-03-03

**Authors:** Soren Ulrik Sonder, Matthew Plassmeyer, Denise Loizou, Oral Alpan

**Affiliations:** Amerimmune, McLean, VA, United States

**Keywords:** basophil activating test (BAT), basophil identification markers, basophil actication test, CBC (complete blood count), flow cytometry

## Abstract

**Background:**

Basophils normally make up <2% of the white blood cells (WBC). There is no clear consensus for basophil identification by flow cytometry. The increased demand for basophil activation test (BAT) to identifying and monitoring allergic patients highlights the need for a standardized approach to identify basophils.

**Methods:**

Using flow cytometry we analyzed whole blood stained with antibodies against: IgE, CD123, CD193, CD203c, CD3, HLADR, FcɛRI, CRTH2 and CD45. We examined unstimulated blood as well as blood stimulated with Anti-IgE and fMLP. Finally, we compared the results to a complete blood count (CBC) from an FDA approved hematological analyzer.

**Results:**

Basophil identification relying on just one surface marker performed worse than approaches utilizing two identification markers. The percentage of basophils from WBC determined by flow cytometry results had a good correlation with the CBC results even though the CBC results were generally higher. Stimulating whole blood with the basophil activators did not interfere with the basophil identification markers.

**Conclusion:**

In flow cytometry assays, two surface markers should be used for identifying basophils and if a very pure basophil fraction is desired a third marker can be considered. In our hands the approaches that included CD123 in combination with either CD193, HLADR^negative^ or FcɛRI performed the best.

## Introduction

The basophils make up <2% of the white blood cells (WBC) in healthy individuals. Historically basophils have not been given much attention. The rapid development in flow cytometry combined with the discovery that CD63 is translocated to the basophil surface after activation lead to the development of the flow cytometry-based basophil activation test (BAT) and has revitalized the interest in basophils (1–5). The BAT is increasing in popularity because it offers a safe alternative to the oral food challenge when diagnosing food allergy (6). Measuring CD63 by flow cytometry has been established as the best marker for basophil activation in the BAT assay. Basophil identification is done with a varsity of markers and no clear standard has been established. It is possible to identify basophils using only one surface marker combined with FSC/SSC but these methods do not always give the best result (7–13). Many clinical and research laboratories use a combination of markers to identify basophils including but not limited to: CD123^+^/CD193^+^, CD123^+^/HLADR^−^, CD3^−^/CD193^+^, CD3^−^/CRTH2^+^, CD193^+^/CD203c^+^ (3, 6, 8, 12, 14–16). In the BAT, basophils go through various stimuli such as anti-IgE and N-Formylmethionyl-leucyl-phenylalanine (fMLP) which are also considered as positive controls for this test, as well as different allergens that are being tested. These stimulations have been reported to affect the expression of several basophil identification markers including CD203c (increased expression), CD123 and CD193 (reduced expression) (16–20). Furthermore, it might not always be possible to process a sample immediately which makes it critical that the identification markers that are chosen to be used in this test are stable *in vitro*. In this study we stain whole blood with an antibody cocktail containing all the above-mentioned antibodies and analyze them by flow cytometry. The aim of this study is to compare the different methods of identifying basophils by flow cytometry. We compare the impact of different gating methods. Furthermore, we compare the expression of the markers after activation with anti-IgE antibody and fMLP. We assessed the post collection stability of the identification markers. Finally, we compare the result for %basophils of WBC with the result of a CBC test obtained using an FDA approved hematology analyzer.

## Methods

### Donors

All clinical investigations were conducted according to Declaration of Helsinki principles. All human studies were approved by the Western Institutional Review Board (IRB 1285028). A total of 79 donors, age range 5 to 87, were utilized for the study. The donors were recruited from patients and employes at Amerimmune LLC, CBC was done by Quest Diagnostics using the FDA approved Sysmex XN11 automated hematology analyzer (Kobe, Japan).

### Basophil phenotyping

Whole blood was collected in a heparin and an EDTA tube. The EDTA tube was used for external CBC analysis. The heparin tube was kept at 18–25°C. Basophil identification was done using unstimulated blood (PBS) as well as blood stimulated with either Anti-IgE-FITC (Thermo Fisher, Waltham, MA) or fMLP (Sigma, St. Louis, MO). The samples were incubated for 20 min at 37°C followed by 10 min at 4°C (4, 14, 21–24). Each sample was stained with the following antibodies anti-IgE-FITC (Clone Ige21), anti-CD193-PE (Clone 5E8), anti-CD123-PerCPCy5.5 (Clone 6H6), anti-CD203c-PECY7 (Clone NP4D6), anti-CRTH2-APC (Clone BM16), anti-CD3-AF700 (Clone UCHT1), anti-CD45-EF506 (Clone HI30) anti-FcɛRI-SB600 (Clone AER-37) (all Thermo Fisher, Waltham, MA) and anti-HLADR-Pacific blue (L243) (Biolegend, San Diego, CA) for 30 min at 4°C. Each antibody was titrated to obtain the best separation (25). The red blood cells were lysed using BD FACS lysis solutions (BD Bioscience, San Jose, CA) and resuspended in 400 μl PBS before acquisition.

### Instrumentation

The samples were acquired on a 3 laser/10 color BD FACSCanto. CS&T beads (BD Bioscience, San Jose, CA) were acquired daily to ensure consistent performance of the cytometer. The instrument has been CAP and CLIA validated for clinical diagnostic studies. All samples were acquired for 5 min at the highest acquisition speed setting.

### Data analysis

**Figure 1:**
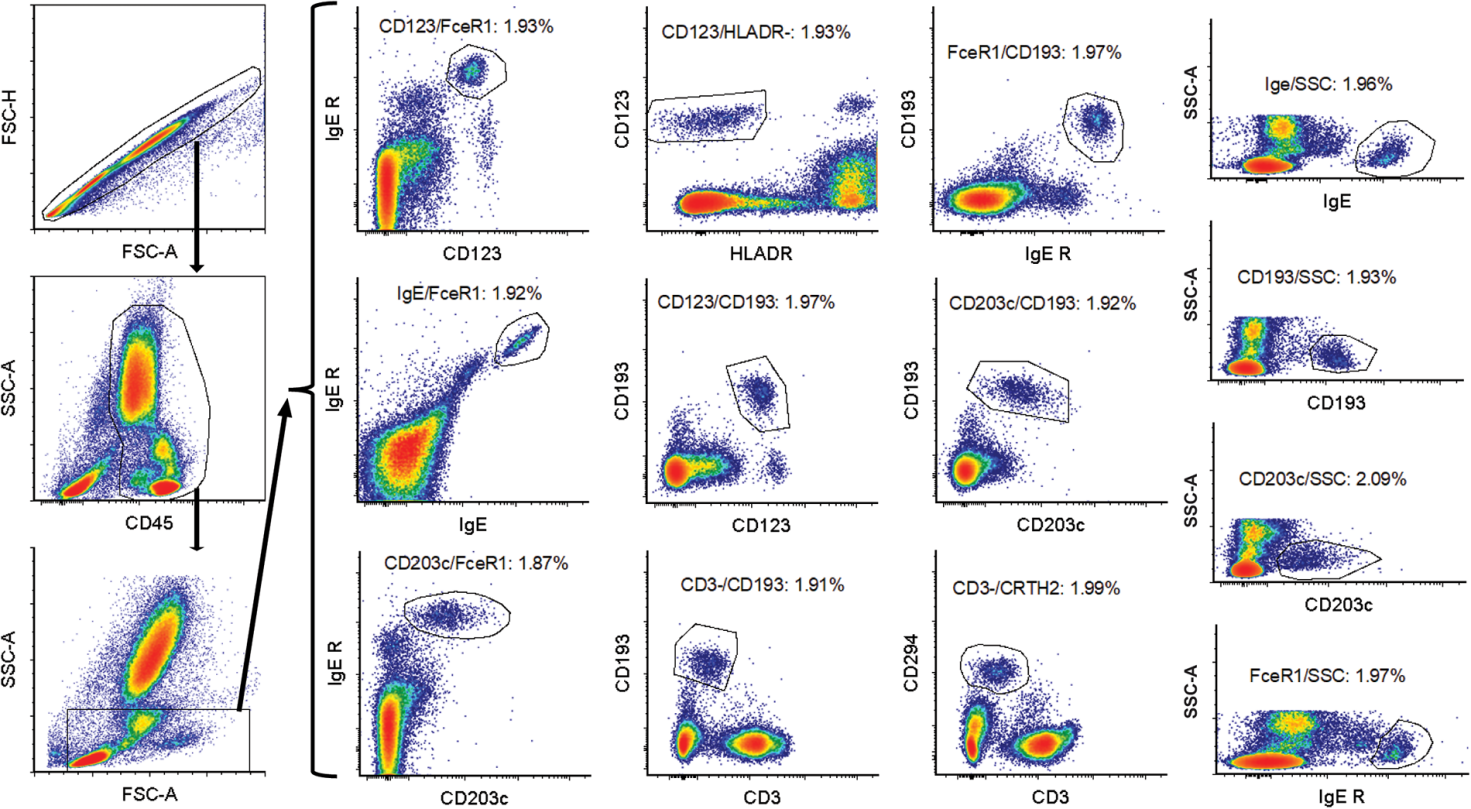
Gating strategy for basophil identification. First an FSC-A/FSC-H gate was used to gate on singlets. Subsequently a CD45/SSC gate was drawn around the white blood cells. This was followed by an FSC-SSC gate to gate out some non-basophils. The basophils were then identified using 13 different gating strategies: CD123/CD193, IgE/FcɛRI, HLADR/CD123, CD203c/FcɛRI, FcɛRI/CD193, CD3/CD294, CD3/CD193, FcɛRI/CD193 and CD123/FcɛRI, IgE/SSC, FcɛRI/SSC, CD193/SSC, and CD203c/SSC.

Data analysis was performed using FCS Express software (De Novo software, Glendale, CA). The gating strategy is to gate on singlets using FSC-A/FSC-H plot. A CD45/SSC plot is used to identify WBC. This is followed by an FSC/SSC gate to gate out eosinophils and majority of neutrophils. The basophils are subsequently identified in 13 different plots: CD123/CD193, IgE/FcɛRI, HLADR/CD123, CD203c/FcɛRI, FcɛRI/CD193, CD3/CD294, CD3/CD193, FcɛRI/CD193, CD123/FcɛRI, IgE/SSC, FcɛRI/SSC, CD193/SSC and CD203c/SSC (Figure 1 and Table 1). The % basophil result is the gated population in each of these plots as percentage of the WBC population.

**Table 1:** Basophil identification markers and flow cytometry parameters used to identify basophils in whole blood samples.

	Marker	Cell expression	Description and gating strategy	References
Basophil identification markers expressed on the cell surface	CD123	Basophils, eosinophils, dendritic cells,	Highly expressed on basophils. Is normally used in combination with another basophil marker or HLADR^negative^.	(8, 17, 26)
	CD193	Basophils, eosinophils, mast cells, Th2 lymphocytes	Solid marker for basophil identification. Have been used alone, in combination with other basophil markers or CD3^negative^.	(8, 10, 12, 27)
	CD294 (CRTH2)	Basophils, eosinophils, T-lymphocytes	Basophils can be differentiated from T-lymphocytes by CD3 and from eosinophils by side scatter	(28, 29)
	IgE	Basophils, monocytes, dendritic cells	Expressed as both a soluble molecule and bound to the FcɛRI on basophils.	(9, 13, 30)
	FcɛRI	Basophils, mast cells, dendritic cells, monocytes in patients with allergic disorder	Bound to IgE. Crosslinking receptors with a relevant allergen of anti-IgE activates the basophil.	(7, 26)
	CD203c	Basophils, CD34+ progenitor cells, mast cells	Used as both an identification and an activation marker for basophils. Is expressed at low levels on resting basophils.	(31)

Graphs were generated as scatter plots, and statistical analysis was performed using GraphPad Prism. All data comparisons were analyzed as paired, two tailed, two-sample unequal variance using the students *t*-test to determine significance. A *p*-value less than 0.05 is considered significant, *p < 0.05, **p < 0.01. Correlation and Bland-Altman analysis and plots were performed using GraphPad Prism (32, 33).

## Results

### Basophil marker stability

Recent published papers by us and other groups show that the BAT is stable up to 28 h post collection (14, 22). The efficacy of this assays as well as other flow cytometry assays involving basophil identification is dependent on stable expression of the chosen markers on the basophils. Table 1 summarizes the different markers and parameters utilized in the study, and the gating strategy is shown in Figure 1.

We started by testing the stability of the different gating strategies to see if the expression of the markers that help identify basophils would change over time. Whole blood was collected in heparin tubes and the expression of the markers were measured by flow cytometry 0–4 h post collection and again after 22–26 h. The blood was stored at room temperature (18–25°C).

**Figure 2:**
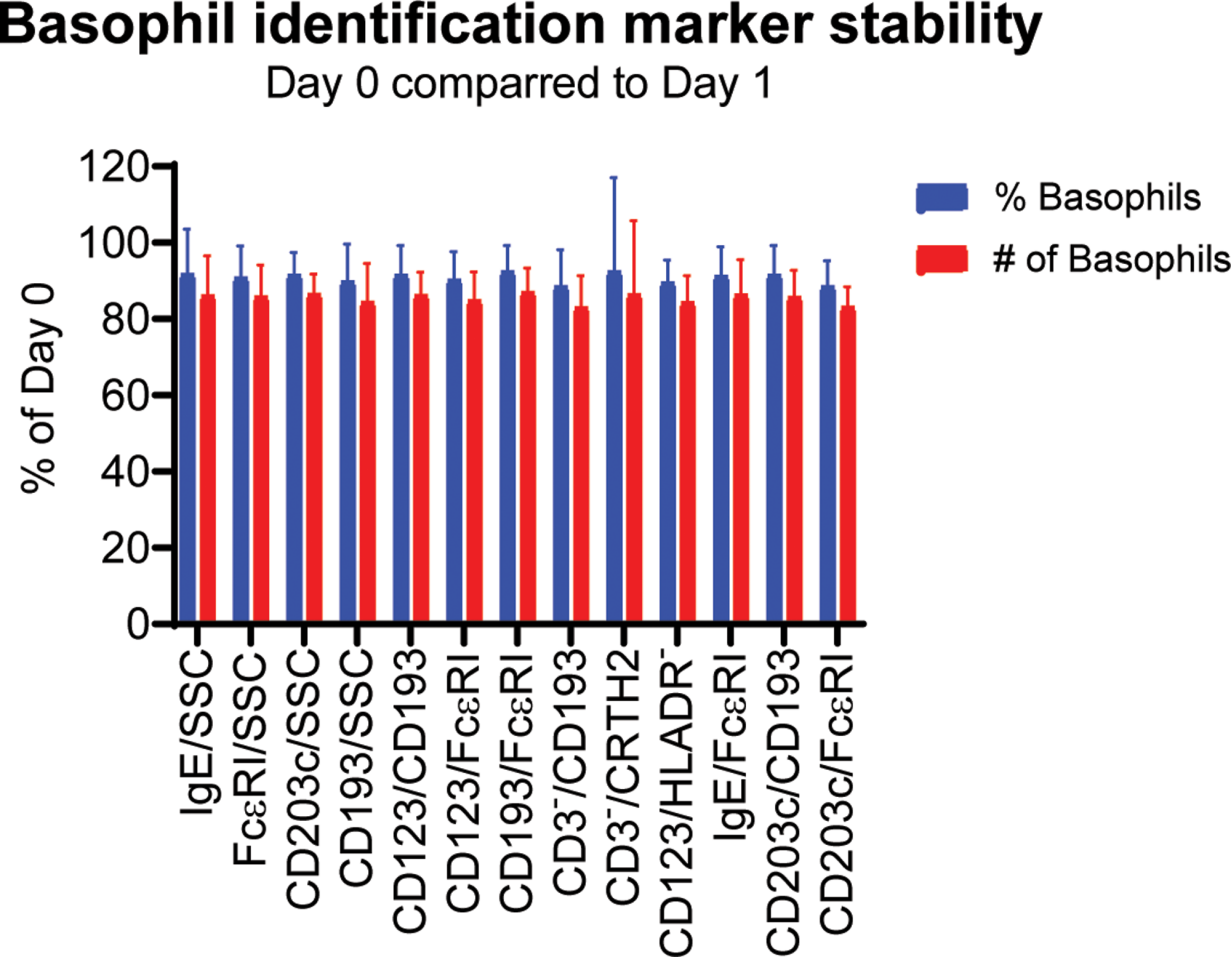
Stability of the different basophil identification markers. Whole blood from 9 donors was collected in heparin tubes and the expression of the markers were measured by flow cytometry after 0–4 h (Day 0) and again after 22–26 h (Day 1) using the gating strategy shown in Figure 1. The blood was stored at room temperature (18–25°C). The % basophils of WBC as well as the total number of basophils identified were measured. The results for Day 0 are set to 100% and the results for Day 1 are normalized accordingly. Student's *t*-test paired, *n* = 9.

The results show a slight reduction in the % basophils to 89%–92% at Day 1 compared to Day 0. The absolute number of basophils collected dropped to 83%–87% of the value at Day 0. The results are very similar for all the tested gating combination with no method detecting a significant different percentages or absolute number of basophils (Figure 2). Based on this experiment we conclude that we can accurately evaluate the percentages of basophils within 22–26 h post collection.

### Frequency of problematic gating

We examined if it was possible to gate on a distinct basophil population with all gating approaches in all the samples. We recorded the instances where the basophil population does not form a separate population and the instances where it was impossible to identify a basophil population at all. Our results show that CD123/CD193 and CD123/HLADR^−^ gave a clear separate population in all analysis. CD203c/FcɛRI, CD193/FcɛRI and CD123/FcɛRI worked for all but one donor. In the other end of the spectrum are IgE/SSC, FcɛRI/SSC and CD3^−^/CDTH2 which frequently could not detect any basophils or often did not give a clear separate population (Table 2). In the patients where it was impossible to identify basophils at all with a given gating combination then the specific combination from that patient was removed from the subsequent analysis.

**Table 2:** Problematic basophil identification.

Issue	Impossible to gate on basophils			Basophils form a shoulder rather than a detached population		
Stimulation	PBS	Anti-IgE	fMLP	PBS	Anti-IgE	fMLP
IgE/SSC	10	10	10	0	0	0
FcɛRI/SSC	7	14	6	7	8	6
CD203c/SSC	1	1	1	3	2	1
CD193/SSC	1	0	1	3	5	8
CD123/CD193	0	0	0	0	0	0
CD123/FcɛRI	0	0	0	0	1	0
CD193/FcɛRI	1	0	0	2	7	4
CD3^−^/CD193	2	0	0	5	8	9
CD3^−^/CRTH2	3	3	2	10	11	14
CD123/HLADR^−^	0	0	0	0	0	0
IgE/FcɛRI	1	1	1	5	3	3
CD203c/CD193	1	1	1	2	1	3
CD203c/FcɛRI	0	0	0	1	0	1

The table show the numbers of samples where it was not possible to identify a basophil population as well as the number of samples where the identification can be prone to error because the basophils does not form a separate population but rather a shoulder on the negative population.

### Correlation between methods the different flow-based methods

Next, we used linear correlation to see if the % basophils of WBC results are similar using the different gating approaches. The R square value ranges from 0.69 (FcɛRI/SSC vs. FcɛRI/IgE) to 0.99 (IgE/SSC vs. CD123/CD193; Ige/SSC vs. CD123/HLADR^−^; IgE/SSC vs. IgE/FcɛRI; and CD123/CD193 vs. CD123/HLADR^−^). To easier appreciate the performance of each gating strategy we calculated the average for the R square values for each gating strategy, The average R square ranking are (high to low): IgE/SSC, CD123/CD193, CD123/FcɛRI, CD123/HLADR^−^ and CD203c/CD193 > CD203c/FcɛRI > IgE/FcɛRI > CD203c/SSC, CD193/FcɛRI, CD3^−^/CRTH2 > CD193/SSC > CD3^−^/CD193 > FcɛRI/SSC. The graphs are all shown in Supplementary Figure S1. The values for the *R* square and the slope are summarized in Table 3.

**Table 3:** Linear correlation between different flow methods.

		*R* square													
		IgE/SSC	FcɛRI/SSC	CD203c/SSC	CD193/SSC	CD123/CD193	CD123/FcɛRI	CD193/FcɛRI	CD3^−^/CD193	CD3^−^/CRTH2	CD123/HLADR^−^	IgE/FcɛRI	CD203c/CD193	CD203c/FcɛRI	Average R square
Slope	IgE/SSC		0.73	0.94	0.92	0.99	0.98	0.91	0.95	0.92	0.99	0.99	0.96	0.97	0.94
	FcɛRI/SSC	0.96		0.72	0.75	0.74	0.79	0.81	0.79	0.78	0.74	0.69	0.79	0.74	0.75
	CD203c/SSC	0.92	0.72	-	0.88	0.94	0.93	0.88	0.91	0.88	0.94	0.90	0.93	0.94	0.90
	CD193/SSC	0.97	0.72	0.99	-	0.95	0.93	0.86	0.90	0.85	0.93	0.89	0.92	0.88	0.89
	CD123/CD193	0.97	0.74	0.99	0.95	-	0.98	0.91	0.95	0.92	0.99	0.95	0.97	0.96	0.94
	CD123/FcɛRI	0.98	0.78	1.01	0.96	1.01	-	0.96	0.95	0.94	0.98	0.93	0.97	0.95	0.94
	CD193/FcɛRI	0.96	0.80	0.98	0.92	0.97	0.99	-	0.91	0.91	0.91	0.89	0.93	0.91	0.90
	CD3^−^/CD193	0.98	0.79	1.00	0.94	0.99	0.98	0.96	-	0.94	0.95	0.92	0.98	0.94	0.85
	CD3^−^/CRTH2	0.94	0.77	0.88	0.90	0.96	0.95	0.93	0.95	-	0.93	0.89	0.93	0.91	0.90
	CD123/HLADR^−^	0.98	0.75	0.96	0.95	1.01	0.98	0.94	0.96	0.97	-	0.96	0.97	0.96	0.94
	IgE/FcɛRI	0.97	0.73	0.98	0.93	0.99	0.96	0.93	0.94	0.96	0.98	-	0.93	0.94	0.91
	CD203c/CD193	0.95	0.76	0.98	0.92	0.97	0.96	0.93	0.96	0.96	0.96	0.94	-	0.96	0.94
	CD203c/FcɛRI	0.91	0.72	0.95	0.87	0.93	0.91	0.89	0.90	0.91	0.93	0.91	0.94	-	0.92

A summary of the linear correlation analysis between the different flow-based basophil identification methods shown in Supplementary Figure S1. Light red background are the R square values, and the light blue background is the slope. The average R square values are the average of the 12 *R* square values where the gating combination on the right is involved.

### Specificity and inclusiveness of the different gating strategies

An essential question is if the basophils identified by one gating strategy will also be identified as basophils by the other gating strategies. We addressed this by displaying the basophils obtained by one gating strategy in each of the 12 other strategies and recording the percentages of the cells that were then identified as basophils. The individual box plots are shown in Supplementary Figure S2 and the mean values are shown in Table 4. The highest specificity defined as the gating method where most of the identified basophils were also identified as basophils in the 12 other approaches is CD123/HLADR^−^ followed by (high to low, as shown in the bottom row in Table 4): CD123/CD193 > IgE/SSC > CD203c/CD193 > CD123/FcɛRI > CD193/FcɛRI > IgE/FcɛRI > CD203c/FcɛRI > CD193/SSC > CD3^−^/CD193 > CD3^−^/CRTH2 > CD203c/SSC > FcɛRI/SSC. This ranking shows how pure the initial captured basophil population is. The summary column on the far right of the table shown the inclusiveness, defined as how well the different gating options are at capturing all the basophil events with the best being IgE/SSC followed by (high lo low): CD123/HLADR^−^ > CD193/FcɛRI > CD123/CD193 > CD123/FcɛRI > CD3^−^/CD193 > CD193/SSC > CD203c/CD193 > IgE/FcɛRI > FcɛRI/SSC > CD3^−^/CRTH2 > CD203c/FcɛRI > CD203c/SSC.

**Table 4:** Linear correlation between different flow methods.

		Initial gate													
		IgE/SSC	FcɛRI/SSC	CD203c/SSC	CD193/SSC	CD123/CD193	CD123/FcɛRI	CD193/FcɛRI	CD3^−^/CD193	CD3^−^/CRTH2	CD123/HLADR^−^	IgE/FcɛRI	CD203c/CD193	CD203c/FcɛRI	Average (Inclusiveness)
% Back-gated	IgE/SSC		74	81	88	94	94	93	86	80	96	98	91	91	88.8
	FcɛRI/SSC	88		75	80	83	86	84	77	72	84	88	81	86	82.0
	CD203c/SSC	82	65		78	81	81	79	74	70	83	79	83	87	78.5
	CD193/SSC	90	70	78		93	88	91	88	79	91	86	91	85	85.8
	CD123/CD193	92	71	80	90		92	92	87	81	95	89	92	88	87.4
	CD123/FcɛRI	94	73	80	86	93		91	84	79	95	92	89	91	87.3
	CD193/FcɛRI	92	73	78	89	94	92		88	81	92	90	92	90	87.6
	CD3^−^/CD193	88	69	76	91	93	88	91		83	90	86	94	85	86.2
	CD3^−^/CRTH2	81	65	71	80	84	82	82	82		84	80	84	79	79.5
	CD123/HLADR^−^	94	72	81	88	95	94	92	86	81		91	91	90	87.9
	IgE/FcɛRI	92	73	75	81	86	88	87	80	76	88		84	87	83.1
	CD203c/CD193	86	67	78	87	90	85	88	86	79	88	84		84	83.5
	CD203c/FcɛRI	84	68	78	77	82	84	82	75	71	83	85	84		79.4
	Average (Specificity)	88.6	70.0	77.6	84.6	89.0	87.8	87.7	82.8	77.7	89.1	87.3	88.0	86.9	

A summary of the linear correlation analysis between the different flow-based basophil identification methods shown in Supplementary Figure S2. Last row with light red background is the average of the 12 values in the column and is a measurement of how specific the gate is for basophils when compared to the 12 other gating methods. The last blue column is the average of each row is the inclusiveness which represents how big percentages of the basophils fall within the given gate.

### Comparing flow cytometry results to CBC

A CBC with differential test is a well-established method that among other results provides the percent basophils of whole blood. For each donor we collected a tube for CBC analysis at an external reference laboratory. The CBC were all run within 24 h of sample collection on an FDA approved hematology analyzer. The linear correlation analysis between the CBC and each of the flow methods show some degree of correlation with an R square value between 0.59 to 0.8. The highest being IgE/SSC, CD193/SSC, CD123/CD193 followed by CD123/FcɛRI, CD123/HLADR- > IgE/FcɛRI, CD203c/FcɛRI > CD203c/CD193 > CD193/FcɛRI > CD203c/SSC > CD3-/CRTH2 > FcɛRI/SSC. The sloop is between 0.74–0.84 indicating that the flow values generally are lower than the CBC values. To better understand the difference between the methods we visualized the data in two different Bland-Altman plots, Difference vs. Average and Ratio vs. Average (32, 33). The results for all flow methods except FcɛRI/SSC show that the CBC systematically are higher than the flow cytometry results. The most pronounced difference is for CBC results between 0.3%–1.3% basophils but even for the higher values the CBC result is higher. The Ratio vs. Average plot show that the CBC values are generally higher by 40%–50% percentages rather than fixed value. The results for the FcɛRI/SSC gating method the diversion between flow cytometry and CBC seems more of a random nature (Table 5 and Supplementary Figure S3).

**Table 5:** Correlation between the different flow cytometry methods of identifying basophils and the results of a CBC obtained using the XN11 automated hematology analyzer at an accredited reference laboratory.

	Correlation analysis		Bland-Altman analysis (Difference vs. Average)		Bland-Altman analysis (Ratio vs. Average)	
	*R* square	Sloop	Bias	SD	Bias	SD
IgE/SSC	0.80	0.82	0.14	0.19	1.5	0.8
FcɛRI/SSC	0.59	0.84	0.02	0.29	1.2	0.6
CD203c/SSC	0.71	0.74	0.14	0.23	1.4	0.6
CD193/SSC	0.80	0.83	0.13	0.18	1.4	0.6
CD123/CD193	0.80	0.81	0.14	0.18	1.5	0.8
CD123/FcɛRI	0.79	0.81	0.13	0.19	1.5	0.8
CD193/FcɛRI	0.74	0.80	0.13	0.21	1.5	0.8
CD3^−^/CD193	0.73	0.79	0.11	0.22	1.4	0.8
CD3^−^/CRTH2	0.67	0.74	0.13	0.24	1.4	0.6
CD123/HLADR^−^	0.79	0.81	0.14	0.19	1.5	0.8
IgE/FcɛRI	0.78	0.81	0.15	0.19	1.6	0.9
CD203c/CD193	0.77	0.78	0.15	0.20	1.5	0.9
CD203c/FcɛRI	0.78	0.76	0.18	0.19	1.6	0.8

The graphs are shown in Supplementary Figure S3.

### Identification of activated basophils

The BAT involves stimulating whole blood with positive controls that activates the basophils in both a IgE/FcɛRI dependent and independent manner. The activation of basophils might affect the expression of the identification markers.

We stimulated whole blood with Anti-IgE-FITC or fMLP before staining for basophil identification and compared it to unstimulated blood (PBS control) to see if stimulation affects our ability to identify basophils.

We observed that fMLP activation decreases CRTH2 expression slightly. Anti-IgE activation reduce the FcɛRI signal. The antibody used for stimulation was the same used for detection which result in an increase in IgE signal after Anti-IgE stimulation. Both anti-IgE and fMLP activation increases CD203c expression. The rest of the markers were unaffected by stimulation (Supplementary Figure S4).

**Figure 3:**
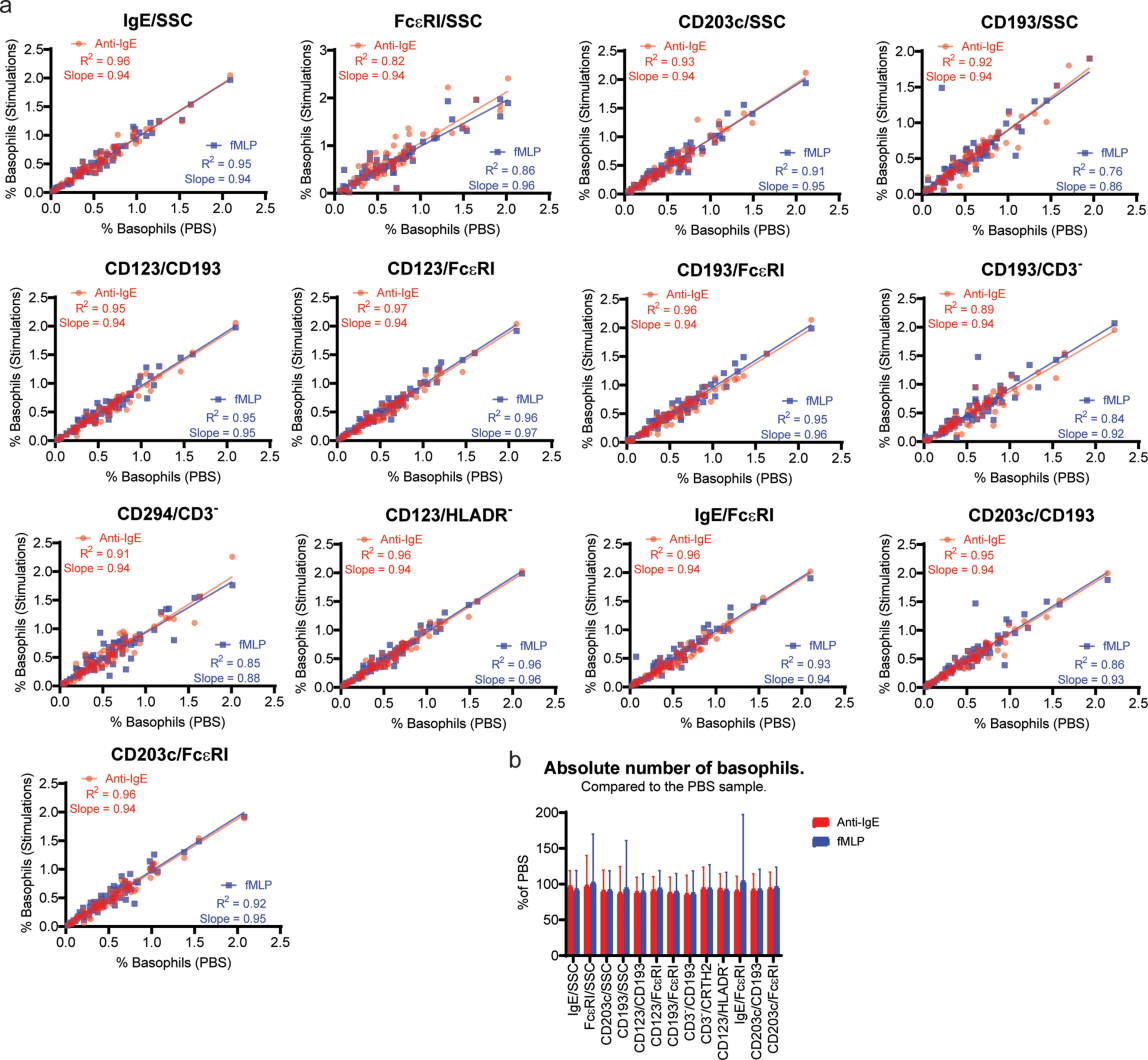
Identification of activated basophils. Whole blood was stimulated with either Anti-IgE FITC or fMLP for 20 min before staining for basophil identification together with the unstimulated control sample (PBS). (**A**) Linear regression analysis shows the comparison between the PBS and Anti-IgE FITC or fMLP for each of the 13 identification methods. The basophils number shown is as % of WBC. (**B**) The absolute number of basophils collected in the Anti-IgE and fMLP stimulated sample using the different gating strategies are shown as percentages of the absolute number of basophils in the PBS sample.

Next, we performed a linear correlation to determine if the percentages of basophil are similar before and after stimulation. The results show an R square above 0.95 for Anti-IgE and fMLP for IgE/SSC, CD123/CD193, CD123/FcɛRI, CD193/FcɛRI, and CD123/HLADR^−^. The lowest correlation with an R square below 0.9 was seen for FcɛRI/SSC, CD193/SSC, CD193/CD3-, CD294/CD3- and CD203c/CD193 (Figure 3A).

Finally, we compared the absolute number of basophils identified to show whether basophil events are gained or lost by stimulating in any of our basophil identification approaches. The results are similar in stimulated and unstimulated samples (Figure 3B).

## Discussion

In this study we examined 13 different gating strategies for basophil identification in a whole blood by flow cytometry. For each gating strategy we looked at (1) Reliability/easiness of gating, (2) Specificity vs. inclusiveness, (3) Correlation between the different approaches, (4) Correlation with CBC results, (5) Stability of the markers, (6) Effect of stimulation.

All the strategies used in this paper to identify basophils utilizing published markers and marker combinations. We started by examining if we get similar results between the different methods. The linear correlation analysis showed the highest correlation between gating strategies utilizing two surface markers unless one of the markers is CD3^negative^ in which case the correlations were low. The single surface markers approaches did not perform well except for IgE/SSC that was among the best. It is worth noting that for 10 of the samples IgE/SSC could not identify any basophils at all. All these patients have elevated levels of circulating IgE that will compete with the surface bound IgE for anti-IgE antibody binding. This approach does not provide us information on the extent the gates captures all the basophils or how basophil specific they are. To understand which strategies are best at capturing the highest percentages of basophils with the least contamination we investigated to what extend a population identified by a given gate would also be identified as basophils using the other gating strategies. The results of this approach are very similar to what we saw in our initial correlation analysis, and it confirms that best gating strategies utilizing two surface markers unless one of the markers is CD3^negative^. It is important to remember that this form of comparison has a bias towards higher specificity and inclusiveness when the same marker is present in both strategies such as comparing CD123/HLADR^negative^ vs. CD123/CD193 or IgE/SSC vs. IgE/FcɛRI.

A selection of hematolytic analyzers is approved by FDA and used at almost all reference laboratories. No flow method for identifying basophils has been FDA approved and we tested to see how well the two approaches compare. Several other studies have compared % basophils from a CBC with flow cytometry and each time the correlation is mediocre with the best *R* square value at 0.68 (8, 27, 34). The correlation in our experiments has an *R* square ranging from 0.59 to 0.80 with seven of our gating strategies having an R square of at least 0.75. The slope ranged from 0.74 to 0.84 showing that the flow result was generally lower than the CBC result. This was confirmed by the Bland Altman plot that showed the CBC to be 40%–50% higher than the flow results with the difference being lower as the basophil percentages increase. The fact that basophil count in a CBC can be overestimated rather than underestimated especially for when the basophil percentages is in or below the normal range has been described previously (34–36).

It has, for the longest time, been a dogma that basophils were unstable cells and that the BAT should be run within 4 h of collecting the sample. Recently studies have shown that the BAT results are stable up to 1 day (20–28 h) post collection (14, 22). Our results show that no matter which gating strategy is used the percentage and absolute number of basophils identified in a 22–26 h old sample is very similar to what can be identified within 4 h post collection. An essential part of the BAT is the stimulation. We did not see any systematically drop in the identified basophils for any of the gating strategies after stimulation with either fMLP or anti-IgE showing that the markers are not shredded or internalized after stimulation to an extend that makes utilization impossible. The gating strategies with the lowest correlation between stimulated and control samples are also the strategies where there were most instances of difficult to identify basophils due to the population being an attached rather than a separate population (Table 2). The strategies with few or no instances of shoulder population had both R square and sloop values very close to 1.

A recurring dilemma in designing a BAT flow panel is on its simplicity and cost-effectiveness. We included CD45 because it is essential for a good WBC gate. We would recommend including it rather than relying solely on FSC and SSC to narrow in on the basophil population.

We do not recommend relying on just one marker for identifying basophils as those strategies did not perform very well in our study. Three of them were not very precise or accurate. IgE/SSC works very well when it does not completely fail which it did more than 13% of the time. Among the gating strategies utilizing two surface markers CD3^−^/CRTH2 and CD3^−^/CD193 performed the worst with respect to inclusiveness and specificity. The IgE/FcɛRI gate has too many instances difficult/impossible to gate. This is typically observed in individuals where the IgE staining does not work. The CD193/FcɛRI approach also have some instances of difficult to gate issues, especially after anti-IgE stimulation, even thou the specificity and inclusiveness is among the best. Not having a well-defined population can cause variation between technologists analyzing the samples and problems if using automatic gating. CD203c expression is low in resting cells and increase after basophil stimulation. This can cause the gate used for identification to shift between samples. Our identification was mainly done on resting cells and for both CD203c/CD193 and CD203c/FcɛRI we saw a high specificity but the inclusiveness we low reflecting that is a problem capturing all the basophils. The remaining three approaches, CD123/CD193; CD123/HLADR^negative^; and CD123/FcɛRI all performs as the best in all our tests, and we recommend choosing one of these. If the flow panel allows for one more parameter, it is possible to combine two methods such as CD123/CD193 and CD123/HLADR^negative^. All three methods include CD123 as one of the parameters and CD123 expression has been reported to be reduced after stimulation (12, 17). This statement has been rebuked by others (37–39). We did not see any reduction in CD123 after stimulation in our experiments. CD123 gave a clear separation in all our experiments but there might be instances where it is not the case, and it will make gating that includes it impossible. Another approach could be to combine one of the CD123 strategies with IgE/SSC. This gate has a very high inclusiveness and specificity but should be excluded from the analysis when a basophil population cannot be identified. If the flow panel allows for four markers to identify basophils it is possible to combine several of the approaches shown here. The advance of using more than one approach is that the gates can be more inclusive, and the specificity can at the same time be increased.

## Data Availability

The original contributions presented in the study are included in the article/**Supplementary Material**, further inquiries can be directed to the corresponding author.

## References

[1] Knol EF Mul FP Jansen H Calafat J Roos D. Monitoring human basophil activation via Cd63 monoclonal antibody 435. J Allergy Clin Immunol. (1991) 88(3 Pt 1):328–38. 10.1016/0091-6749(91)90094-5 1716273

[2] Boumiza R Debard AL Monneret G. The basophil activation test by flow cytometry: recent developments in clinical studies, standardization and emerging perspectives. Clin Mol Allergy. (2005) 3:9. 10.1186/1476-7961-3-9 15989690 PMC1190199

[3] Hoffmann HJ Knol EF Ferrer M Mayorga L Sabato V Santos AF Pros and cons of clinical basophil testing (bat). Curr Allergy Asthma Rep. (2016) 16(8):56. 10.1007/s11882-016-0633-6 27411319

[4] Hoffmann HJ Santos AF Mayorga C Nopp A Eberlein B Ferrer M The clinical utility of basophil activation testing in diagnosis and monitoring of allergic disease. Allergy. (2015) 70(11):1393–405. 10.1111/all.12698 26198455

[5] Santos AF Shreffler WG. Road map for the clinical application of the basophil activation test in food allergy. Clin Exp Allergy. (2017) 47(9):1115–24. 10.1111/cea.12964 28618090 PMC5601249

[6] Santos AF Alpan O Hoffmann HJ. Basophil activation test: mechanisms and considerations for use in clinical trials and clinical practice. Allergy. (2021) 76(8):2420–32. 10.1111/all.14747 33475181

[7] Behrends J Schwager C Hein M Scholzen T Kull S Jappe U. Innovative robust basophil activation test using a novel gating strategy reliably diagnosing allergy with full automation. Allergy. (2021) 76(12):3776–88. 10.1111/all.14900 33973252

[8] Kim Z Choi BS Kim JK Won DI. Basophil markers for identification and activation in the indirect basophil activation test by flow cytometry for diagnosis of autoimmune Urticaria. Ann Lab Med. (2016) 36(1):28–35. 10.3343/alm.2016.36.1.28 26522756 PMC4697340

[9] Stone KD Prussin C Metcalfe DD. Ige, mast cells, basophils, and eosinophils. J Allergy Clin Immunol. (2010) 125(2 Suppl 2):S73–80. 10.1016/j.jaci.2009.11.017 20176269 PMC2847274

[10] Hausmann OV Gentinetta T Fux M Ducrest S Pichler WJ Dahinden CA. Robust expression of Ccr3 as a single basophil selection marker in flow cytometry. Allergy. (2011) 66(1):85–91. 10.1111/j.1398-9995.2010.02431.x 20608915

[11] Eberlein B León Suárez I Darsow U Ruëff F Behrendt H Ring J. A new basophil activation test using Cd63 and Ccr3 in allergy to antibiotics. Clin Exp Allergy. (2010) 40(3):411–8. 10.1111/j.1365-2222.2009.03426.x 20082620

[12] Eberlein B Hann R Eyerich S Pennino D Ring J Schmidt-Weber CB Optimizing of the basophil activation test: comparison of different basophil identification markers. Cytometry B Clin Cytom. (2015) 88(3):183–9. 10.1002/cyto.b.21203 25399741

[13] Abuaf N Rostane H Rajoely B Gaouar H Autegarden JE Leynadier F Comparison of two basophil activation markers Cd63 and Cd203c in the diagnosis of amoxicillin allergy. Clin Exp Allergy. (2008) 38(6):921–8. 10.1111/j.1365-2222.2008.02960.x 18331364

[14] Kim T Yu J Li H Scarupa M Wasserman RL Economides A Validation of inducible basophil biomarkers: time, temperature and transportation. Cytometry B Clin Cytom. (2021) 100(6):632–44. 10.1002/cyto.b.21991 33539657 PMC9291082

[15] Hemmings O Kwok M McKendry R Santos AF. Basophil activation test: old and new applications in allergy. Curr Allergy Asthma Rep. (2018) 18(12):77. 10.1007/s11882-018-0831-5 30430289 PMC6244909

[16] Monneret G. Ccr3 for basophil activation test: a necessary but insufficient step. Clin Exp Allergy. (2010) 40(6):953; author reply 4. 10.1111/j.1365-2222.2010.03516.x 20557552

[17] Santos AF Bécares N Stephens A Turcanu V Lack G. The expression of Cd123 can decrease with basophil activation: implications for the gating strategy of the basophil activation test. Clin Transl Allergy. (2016) 6:11. 10.1186/s13601-016-0100-4 27042292 PMC4818434

[18] Chirumbolo S. Major pitfalls in bat performance may be caused by gating protocols and Cd63% cut off evaluation. Cytometry A. (2014) 85(5):382–5. 10.1002/cyto.a.22466 24753185

[19] Bühring HJ Streble A Valent P. The basophil-specific ectoenzyme E-Npp3 (Cd203c) as a marker for cell activation and allergy diagnosis. Int Arch Allergy Appl Immunol. (2004) 133(4):317–29. 10.1159/000077351 15031605

[20] Khanolkar A Burden SJ Hansen B Wilson AR Philipps GJ Hill HR. Evaluation of Ccr3 as a basophil activation marker. Am J Clin Pathol. (2013) 140(3):293–300. 10.1309/ajcplsn0rqkhjx1a 23955446

[21] Sturm GJ Kranzelbinder B Sturm EM Heinemann A Groselj-Strele A Aberer W. The basophil activation test in the diagnosis of allergy: technical issues and critical factors. Allergy. (2009) 64(9):1319–26. 10.1111/j.1398-9995.2009.02004.x 19243362

[22] Mukai K Gaudenzio N Gupta S Vivanco N Bendall SC Maecker HT Assessing basophil activation by using flow cytometry and mass cytometry in blood stored 24 hours before analysis. J Allergy Clin Immunol. (2017) 139(3):889–99.e11. 10.1016/j.jaci.2016.04.060 27527263 PMC5237629

[23] Sousa N Martinez-Aranguren R Fernandez-Benitez M Ribeiro F Sanz ML. Comparison of basophil activation test results in blood preserved in acid citrate dextrose and edta. J Investig Allergol Clin Immunol. (2010) 20(6):535–6. PMID:. 21243941

[24] Ebo DG Elst J van Houdt M Pintelon I Timmermans JP Horiuchi T Flow cytometric basophil activation tests: staining of exteriorized basophil granule matrix by fluorescent avidin versus appearance of Cd63. Cytometry B Clin Cytom. (2020) 98(6):483–90. 10.1002/cyto.b.21868 32012452

[25] Ryherd M Plassmeyer M Alexander C Eugenio I Kleschenko Y Badger A Improved panels for clinical immune phenotyping: utilization of the violet Laser. Cytometry B Clin Cytom. (2017) 94(5):671–79. 10.1002/cyto.b.21532 28493330

[26] Duriancik DM Hoag KA. Mistaken identity: purified basophils likely contaminated with dendritic cells. Cytometry A. (2014) 85(7):570–2. 10.1002/cyto.a.22476 24757016

[27] Ducrest S Meier F Tschopp C Pavlovic R Dahinden CA. Flowcytometric analysis of basophil counts in human blood and inaccuracy of hematology analyzers. Allergy. (2005) 60(11):1446–50. 10.1111/j.1398-9995.2005.00910.x 16197480

[28] Monneret G. Is this time for Crth2/Dp2 in a flow cytometric basophil activation test? Clin Exp Allergy. (2008) 38(7):1239–40. 10.1111/j.1365-2222.2008.03021.x 18477015

[29] Nagata K Hirai H Tanaka K Ogawa K Aso T Sugamura K Crth2, an orphan receptor of T-helper-2-cells, is expressed on basophils and eosinophils and responds to mast cell-derived factor(S). FEBS Lett. (1999) 459(2):195–9. 10.1016/s0014-5793(99)01251-x 10518017

[30] Gane P Pecquet C Lambin P Abuaf N Leynadier F Rouger P. Flow cytometric evaluation of human basophils. Cytometry. (1993) 14(3):344–8. 10.1002/cyto.990140316 8472612

[31] Hauswirth AW Natter S Ghannadan M Majlesi Y Schernthaner GH Sperr WR Recombinant allergens promote expression of Cd203c on basophils in sensitized individuals. J Allergy Clin Immunol. (2002) 110(1):102–9. 10.1067/mai.2002.125257 12110828

[32] Bland JM Altman DG. Statistical methods for assessing agreement between two methods of clinical measurement. Lancet (London, England). (1986) 1(8476):307–10. 10.1016/S0140-6736(86)90837-8 2868172

[33] Giavarina D. Understanding bland altman analysis. Biochem Med (Zagreb). (2015) 25(2):141–51. 10.11613/bm.2015.015 26110027 PMC4470095

[34] Amundsen EK Henriksson CE Holthe MR Urdal P. Is the blood basophil count sufficiently precise, accurate, and specific?: three automated hematology instruments and flow cytometry compared. Am J Clin Pathol. (2012) 137(1):86–92. 10.1309/ajcp19bfthytmoro 22180481

[35] Geneviève F Godon A Marteau-Tessier A Zandecki M. Automated hematology analysers and spurious counts part 2. Leukocyte count and differential. Ann Biol Clin. (2012) 70(2):141–54. 10.1684/abc.2012.0665 22484525

[36] Feriel J Depasse F Geneviève F. How I investigate basophilia in daily practice. Int J Lab Hematol. (2020) 42(3):237–45. 10.1111/ijlh.13146 31841278

[37] Chirumbolo S. Commentary: the expression of Cd123 can decrease with basophil activation: implications for the gating strategy of the basophil activation test. Front Immunol. (2016) 7:260. 10.3389/fimmu.2016.00260 27456009 PMC4935682

[38] Chirumbolo S Ortolani R Vella A. Ccr3 as a single selection marker compared to Cd123/hladr to isolate basophils in flow cytometry: some comments. Cytometry A. (2011) 79(2):102–6. 10.1002/cyto.a.21008 21265004

[39] Chirumbolo S Vella A Ortolani R De Gironcoli M Solero P Tridente G Differential response of human basophil activation markers: a multi-parameter flow cytometry approach. Clin Mol Allergy. (2008) 6:12. 10.1186/1476-7961-6-12 18925959 PMC2584049

