# Supplementary material for: Towards standardizing basophil identification by flow cytometry
Source: Front Allergy. 2023 Mar 3;4:1133378. doi: 10.3389/falgy.2023.1133378 (PMC10020589; doi:10.3389/falgy.2023.1133378)
Supplement: Supplementary file 1 [file Datasheet1.docx]

Supplemental figure legends

Supplemental figure S1

The plot shows the correlation between percentage basophils of WBC identified by the different flow cytometry gating methods. The samples where it was not possible to identify basophils in one of the gating schemes have been removed from the relevant correlations. The R square, slope, n value and y-intercept are shown on each graph. Individual linear correlation plots are summarized in Table 3.

Supplemental figure S2

Consistency between different gating methods. The basophils identified by one of the 13 methods shown in figure 1 were backgated to each of the other 12 basophil identification gates. The boxplots show the percentage of the cells initially identified as basophils were identified as basophils by back gating with the other 12 basophil gating schemes shown on the x-axis of each plot. The whiskers show 5-95% confidence interval. Outliers are shown individually. The relevant n-values are shown above each plot. The mean values are shown in Table 4.

Supplemental figure S3

Show the linear correlation and the Bland-Altman plot (Ratio vs Average and Difference vs Average) for the comparison of the %basophils obtained by CBC at an external reference lab (Quest Diagnostics) with the 13 different flow cytometry gating strategies. The results are summarized in Table 5.

Supplemental figure S4

Effect of stimulation on expression of the different identification markers. The initial gating was done as in figure 1. The quadrant is placed next to the population of interest in the PBS (unstimulated) sample and copied to the exact same spot on the plots for the two stimulated conditions (anti-IgE and fMLP).

Supplemental figure S1

|  |  |  |
| --- | --- | --- |
|  |  |  |
|  |  |  |
|  |  |  |
|  |  |  |
|  |  |  |
|  |  |  |
|  |  |  |
|  |  |  |
|  |  |  |
|  |  |  |
|  |  |  |
|  |  |  |
|  |  |  |
|  |  |  |
|  |  |  |
|  |  |  |
|  |  |  |
|  |  |  |
|  |  |  |
|  |  |  |
|  |  |  |
|  |  |  |
|  |  |  |
|  |  |  |
|  |  |  |

Supplemental figure S2

|  |  |
| --- | --- |
|  |  |
|  |  |
|  |  |
|  |  |
|  |  |
|  |  |

Supplemental figure S3

| Bland-Altman difference vs average plots | Bland-Altman ratio vs average plots | Linear correlation plots |
| --- | --- | --- |
|  |  |  |
|  |  |  |
|  |  |  |
|  |  |  |
|  |  |  |
|  |  |  |
|  |  |  |
|  |  |  |
|  |  |  |
|  |  |  |
|  |  |  |
|  |  |  |
|  |  |  |

Supplemental figure S4

| Gate | PBS (unstimulated) | Anti-IgE stimulation | fMLP stimulation |
| --- | --- | --- | --- |
| IgE/  SSC | 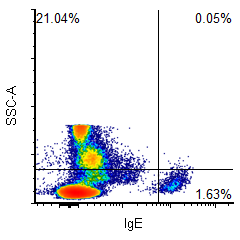 | 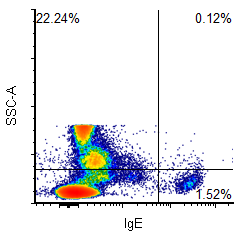 | 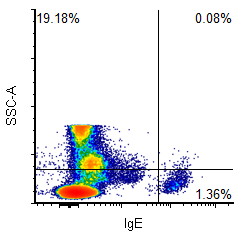 |
| FcεRI/  SSC | 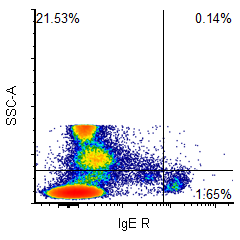 | 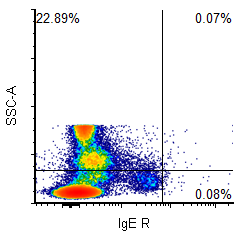 | 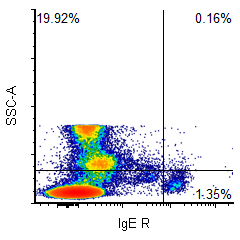 |
| CD203c/  SSC | 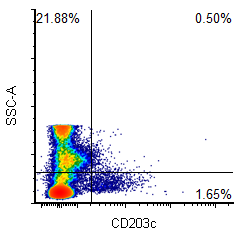 | 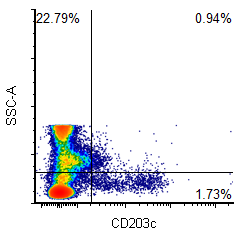 | 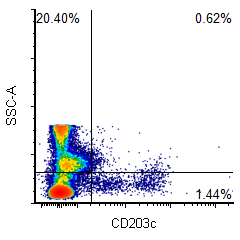 |
| CD193/  SSC | 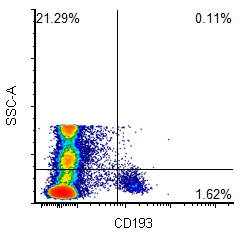 | 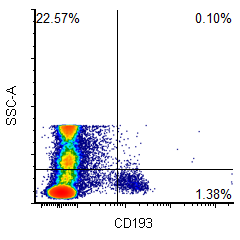 | 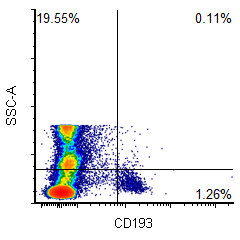 |
| CD123/  CD193 | 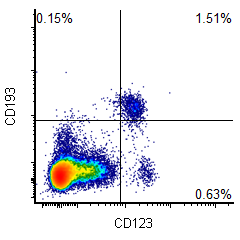 | 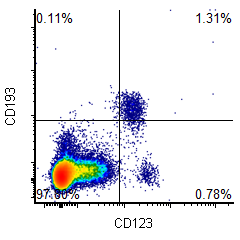 | 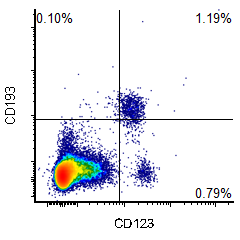 |
| CD123/  FcεRI | 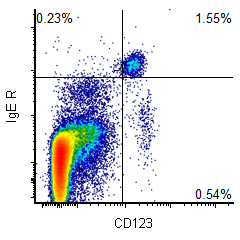 | 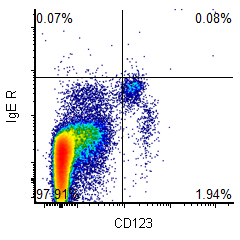 | 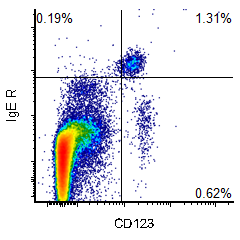 |
| CD193/  FcεRI | 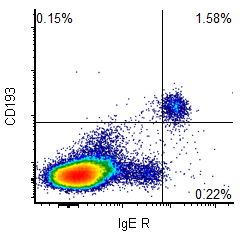 | 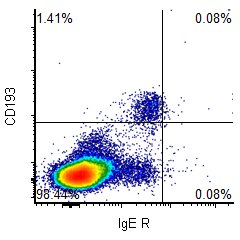 | 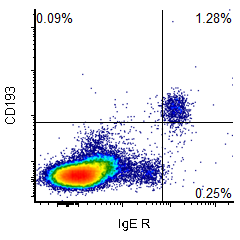 |
| CD3^-^/  CD193 | 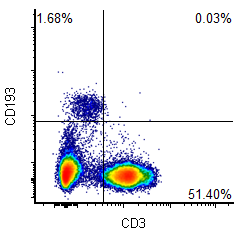 | 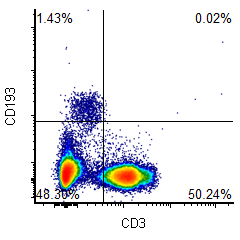 | 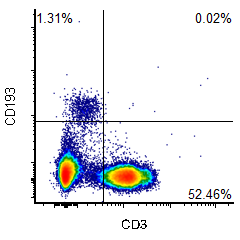 |
| CD3^-^/  CRTH2 | 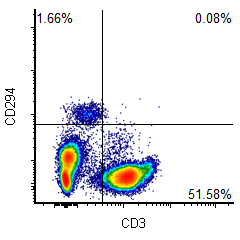 | 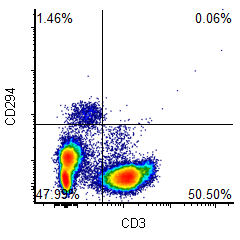 | 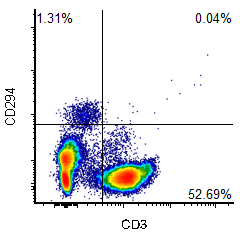 |
| CD123/  HLADR^-^ | 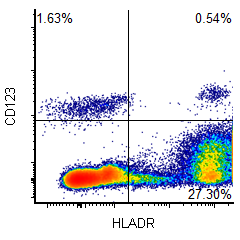 | 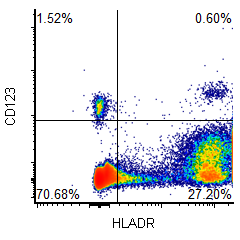 | 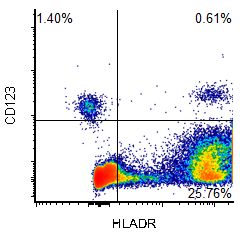 |
| IgE/  FcεRI | 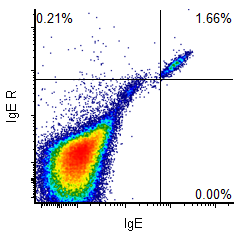 | 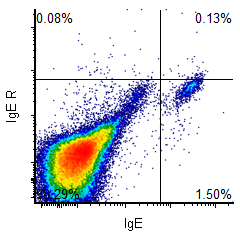 | 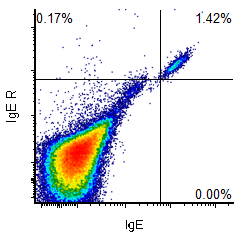 |
| CD203c/  CD193 | 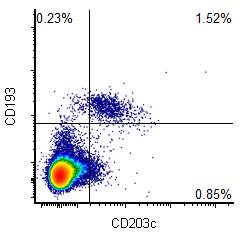 | 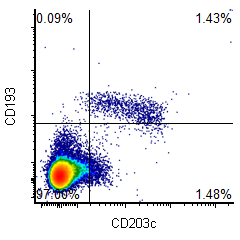 | 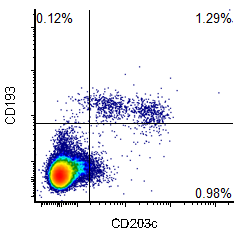 |
| CD203c/  FcεRI | 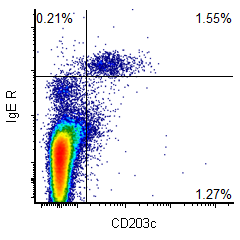 | 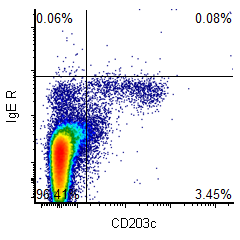 | 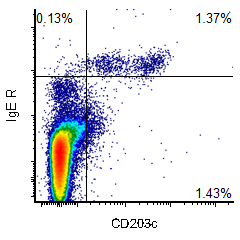 |
